# Economic evaluation of a mentorship and enhanced supervision program to improve quality of integrated management of childhood illness care in rural Rwanda

**DOI:** 10.1371/journal.pone.0194187

**Published:** 2018-03-16

**Authors:** Anatole Manzi, Jean Claude Mugunga, Hari S. Iyer, Hema Magge, Fulgence Nkikabahizi, Lisa R. Hirschhorn

**Affiliations:** 1 University of Rwanda, College of Medicine and Health Sciences; Kigali, Rwanda; 2 Partners In Health; Kigali; Rwanda and Boston, United States of America; 3 Division of Global Health Equity, Brigham and Women’s Hospital; Boston, United States of America; 4 Department of Epidemiology, Harvard T. H. Chan School of Public Health; Boston, United States of America; 5 Division of General Pediatrics, Boston Children’s Hospital; Boston, United States of America; 6 Rwanda Ministry of Health; Kigali, Rwanda; 7 Department of Global Health and Social Medicine, Harvard Medical School; Boston, United States of America; 8 Ariadne Labs, Boston, United States of America; University of Waterloo, CANADA

## Abstract

**Background:**

Integrated management of childhood illness (IMCI) can reduce under-5 morbidity and mortality in low-income settings. A program to strengthen IMCI practices through Mentorship and Enhanced Supervision at Health centers (MESH) was implemented in two rural districts in eastern Rwanda in 2010.

**Methods:**

We estimated cost per improvement in quality of care as measured by the difference in correct diagnosis and correct treatment at baseline and 12 months of MESH. Costs of developing and implementing MESH were estimated in 2011 United States Dollars (USD) from the provider perspective using both top-down and bottom-up approaches, from programmatic financial records and site-level data. Improvement in quality of care attributed to MESH was measured through case management observations (n = 292 cases at baseline, 413 cases at 12 months), with outcomes from the intervention already published. Sensitivity analyses were conducted to assess uncertainty under different assumptions of quality of care and patient volume.

**Results:**

The total annual cost of MESH was US$ 27,955.74 and the average cost added by MESH per IMCI patient was US$1.06. Salary and benefits accounted for the majority of total annual costs (US$22,400 /year). Improvements in quality of care after 12 months of MESH implementation cost US$2.95 per additional child correctly diagnosed and $5.30 per additional child correctly treated.

**Conclusions:**

The incremental costs per additional child correctly diagnosed and child correctly treated suggest that MESH could be an affordable method for improving IMCI quality of care elsewhere in Rwanda and similar settings. Integrating MESH into existing supervision systems would further reduce costs, increasing potential for spread.

## Introduction

Integrated Management of Childhood Illness (IMCI) has been accepted as an effective intervention to improve quality of under-5 care in Sub-Saharan Africa [1–3]. While the success of IMCI in improving under-5 care has been demonstrated in large observational and smaller facility-based studies [4–10], health systems in low-resource settings continue to face challenges in delivering high quality IMCI services [6,11–15]. Sustaining high quality delivery of IMCI services following provider training is often difficult due to inadequate health provider performance [16], high training costs [17], lack of essential medicines and equipment [18], and limited post-training follow-up and supervision [18].

Rwanda implemented IMCI in 2006 with challenges similar to those reported in other countries. To address the gap in quality of care for IMCI, Partners in Health (PIH) in collaboration with the Ministry of Health (MoH) implemented the Mentorship and Enhanced Supervision at Health Centers (MESH) program in two rural districts, with the ultimate goal to provide ongoing post-training mentorship and strengthen data use for ongoing quality improvement [19]. IMCI was one of the four clinical areas that MESH supported, and we refer to this program component as MESH-IMCI. An earlier evaluation demonstrated effectiveness of this program to improve correct classification of under-5 illness and correct treatment administered to children visiting supported health centers [19].

While economic evaluations have been conducted to determine the cost-effectiveness of IMCI interventions on reducing under-5 mortality [10], most have focused on the training component of IMCI, rather than the costs associated with sustaining quality IMCI coverage.

This study estimates the annual cost of implementing MESH-IMCI, and provides estimates of the cost per child correctly diagnosed and the cost per child correctly treated.

Our study augments previous evaluations of IMCI implementation by providing cost estimates for a post-training, health center-focused IMCI quality improvement program. The findings from this economic evaluation can help inform managers and policy makers seeking to allocate resources and set priorities in under-5 health considering the use of use the MESH model as a strategy to improve IMCI-delivery at rural health centers (HCs) in resource-limited settings [20].

## Methods

### Study setting, population and intervention

**Fig 1:**
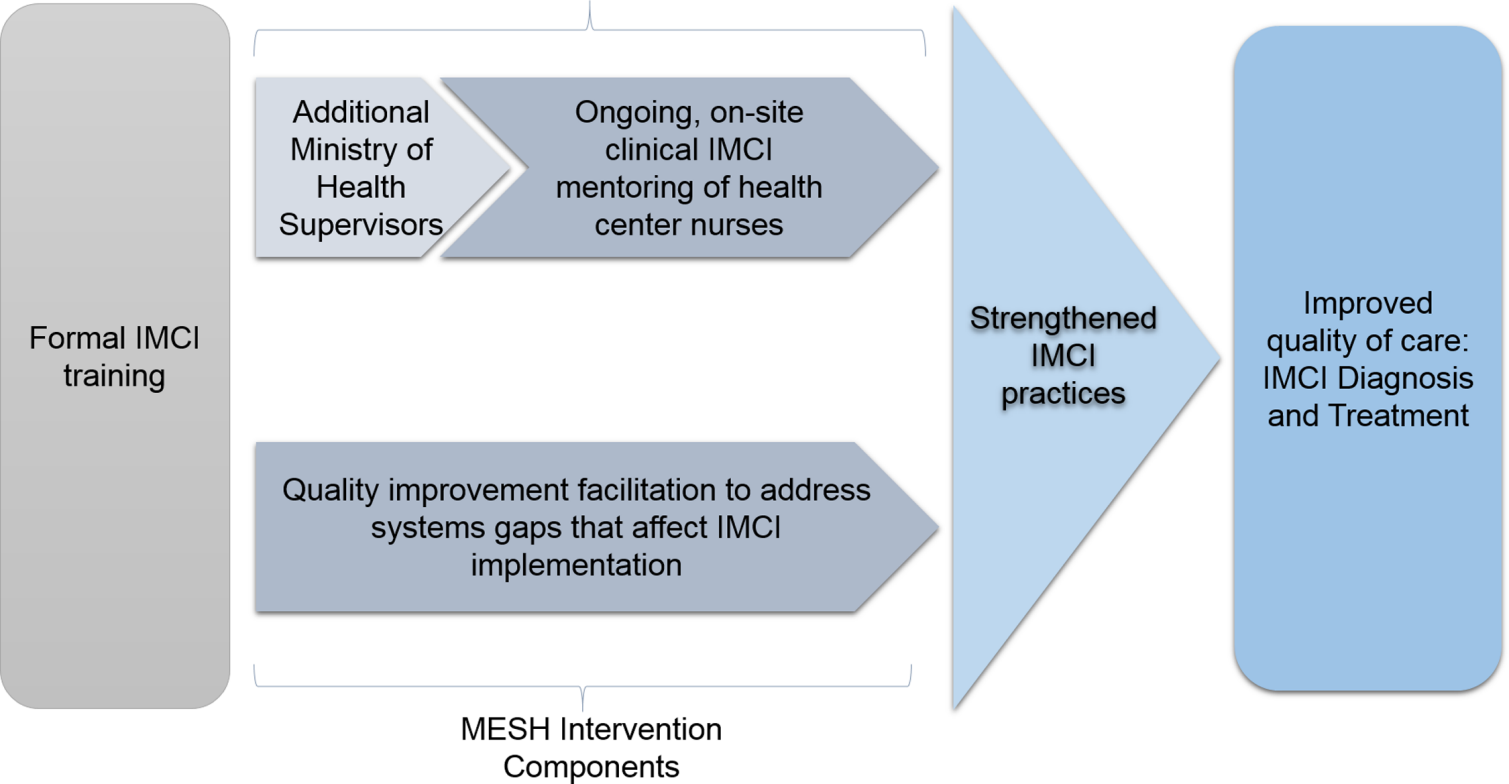
Mentorship and enhanced supervision at health centers: Conceptual framework.

The MESH-IMCI intervention has been described elsewhere [19] and was developed as a component of the Doris Duke Charitable Foundation Africa Health Initiative-funded health systems strengthening project in two districts in rural Rwanda [13]. Briefly, the intervention consisted of 1) day-long scheduled visits every four to six weeks by mentors to health centers, where they provided coaching of HC nurses in clinical care delivery and quality improvement practices using observation data collected during visits, and 2) monitoring and evaluation (M&E) of quality of care data (Fig 1).

MESH-IMCI covered all 21 health centers (HCs) in southern Kayonza and Kirehe districts of Rwanda, covering a catchment of about 530,000 people [21]. Sixty-three participating nurses received mentorship by one of two IMCI-trained MESH mentors. Clinical mentors were MoH-employed expert nurses with university degrees and extensive experience in IMCI care delivery. MESH was sequentially introduced to HCs from December 2010 to May 2011 period, and the post-implementation evaluation was conducted 12 to 14 months following initial implementation [19]. All HCs are run by the Rwandan MoH, with technical and financial support provided by PIH. The HCs had received targeted financial support to improve infrastructure, availability of essential medicines and equipment, and M&E systems as an earlier component of the health systems strengthening initiative, allowing MESH-IMCI to focus on supporting the health providers at facilities to deliver high quality under-5 care [13,22].

As typical Rwandan HCs, in addition to IMCI, as strategy for primary care delivery, targeted facilities covered three main categories of health services including 1) promotional activities which include information, education, and communication; 2) psychosocial support; nutritional activities; community engagement; management and financing of health services; home visits; and hygiene and sanitation in the catchment area around the health center; and 3) preventive activities including premarital consultation, antenatal care, postpartum and newborn care, family planning, school health, epidemiologic surveillance activities and outreach activities such as vaccinations [23].

### Data collection

#### Correct treatment and classification of IMCI patients

The evaluation used a pre/post design in which 292 observations were conducted at baseline and 413 were at 12 months [19]. Quality of care data were captured by observers using case observation checklists developed from the World Health Organization (WHO) and MoH tools [24]. Outcomes were based on validated measures from the IMCI multi-country evaluation (MCE) [25]. The difference in proportion of children correctly diagnosed and the proportion of children correctly treated according to IMCI protocols from baseline to twelve months was used to estimate the change in effectiveness of IMCI care.

#### Cost data

MESH-IMCI program cost data were obtained from program budgets and financial records during the implementation period (fiscal year 2012) [26] as part of an overall costing study integrated into the DDCF AHI project. Data on additional resources used for implementation of MESH-IMCI (e.g. administrative costs for human resources and financial support, logistics and transportation, M&E costs) were obtained using PIH’s annual expenditure reports for that time period.

#### Health utilization data

In order to estimate per-visit costs associated with MESH-IMCI, we obtained monthly under-5 visit data for the 21 facilities in the MESH-IMCI intervention area from Rwanda’s Health Management Information System, an electronic database that tracks monthly service utilization from all public facilities in the country with high data quality during the study period [27].

### Data analysis

#### Program costs

Total costs of the MESH-IMCI program were estimated using the six-step approach developed by Larson et al. [28]. The six-step approach details how to estimate program costs using existing financial records, developing input categories, estimating donations and overhead costs, and estimating annual equivalent payments for large purchases. We defined six major cost categories (salaries, quality improvement trainings, M&E, mentoring visits, and data review and prioritization sessions and overhead). Salary costs included costs related to mentor and manager salaries, communication, and per diems. Quality improvement training costs included costs for renting space, materials for training, and food for attendees. These costs were amortized using a 10% discount rate over three years. M&E costs included costs for data entry, data quality checks, and generation of quarterly reports. Mentoring visit costs included costs of transport to and from facilities for mentors.

Data review and prioritization activities included costs associated with invited stakeholder workshops. Overhead costs included costs of using human resource and finance team time at PIH for MESH-IMCI. Since IMCI was one of four clinical areas that MESH supported [29], we apportioned programmatic costs to MESH-IMCI based on interviews with MESH program managers and program activity logs (roughly 25% of total costs).

#### Cost per visit

To estimate the cost of MESH-IMCI per visit we divided the total annual costs of the program by the annual number of IMCI consultations in fiscal year 2012. Costs were initially collected in Rwandan Francs (FRW) and converted to 2011 US Dollars (USD) using a rate of 604RWF per 1USD, the median exchange rate for fiscal year 2011 [30]. To measure the fixed costs for equipment, upfront investment costs were estimated using a replacement cost approach and annualized using a 3% discount rate and an estimated working life (5 years for most of the equipment).

We conducted our analysis using the district health system (provider) perspective.

While this perspective is narrower than the societal perspective, we elected to use it due to time and resource limitations. Further, we considered it as sufficient to assess the relevant costs and effectiveness of interest to managers and policy makers responsible for planning and for scale-up.

We excluded the following from our estimates because they are included in IMCI nationally mandated training and provision of IMCI-directed services: 1) costs for IMCI care beyond the level of HCs (e.g., referrals to district hospitals, care in the community), 2) costs of medical equipment and space at health centers required to conduct IMCI consults, and 3) costs to the patients (e.g., insurance and co-payment fees, transport, caregiver time during the child visit), 4) cost of mentored nurse time, 5) medicines administered at IMCI visit, and 6) costs of IMCI trainings for HC nurses.

#### Estimating cost per child correctly diagnosed and cost per child correctly treated

We estimated the cost per child correctly diagnosed and the cost per child correctly treated by comparing costs and outcomes of a simulated population of 1,000 children who received usual care and 1,000 children who received care supported by MESH-IMCI. We assumed that the proportion of children correctly diagnosed and treated through usual care would be the same as the baseline care measured in the MESH-IMCI evaluation and similarly the proportion receiving current diagnosis and treatment through IMCI-MESH supplemented care would be the same as the end-line data. The incremental cost-effectiveness ratio (ICER) was defined as the incremental cost per percentage increase quality based on the two process indicators (diagnosis and treatment). ICER was calculated as the ratio of the difference in the total cost per IMCI visit before and after the implementation of MESH, and the difference in quality improvement (correctness of the diagnosis and treatment). As such, *ICER = (Cb—Ce) / Eb—Ee)*; where: *Cb* is the cost at the baseline before MESH; *Ce* is the cost at the end-line (12 months into the implementation of MESH); *Eb* is the percentage of quality of care at the baseline and Ee is the percentage improvement in quality of care after MESH.

To account for uncertainty in our estimates of the cost per child correctly treated and cost per child correctly diagnosed, we ran a sensitivity analysis assuming different parameters that could affect these estimates: 1) quality of care at baseline, and 2) patient volume. In each scenario, we calculated the incremental cost effectiveness ratio (ICER) per patient correctly diagnosed and patient correctly treated. We modeled the percentages of improvement in quality of care (correct diagnosis and correct treatment) per 1000 patients to determine the cost-effectiveness ratio for both the correct classification and correct treatment.

### Ethics statement

This project was approved by the Partners Institutional Review Board, Boston, USA and the Rwanda National Ethics Committee, Kigali, Rwanda.

## Results

Annual MESH-IMCI intervention costs were estimated to be $27,955.74 (Table 1). Salaries for mentors and program staff accounted for the largest proportion of MESH-IMCI costs (80%), while costs of M&E of the program accounted for about 7% of the total. The initial mentor orientation training in clinical mentoring and quality improvement (QI) techniques accounted for 1% of the total intervention costs.

**Table 1:** Distribution of annual MESH IMCI program costs.

Input category	Cost (2011 USD)	Cost percent
MESH salary and benefits	$ 22,400.00	80%
MESH Quality Improvement trainings	$ 239.36	1%
Monitoring and Evaluation	$ 2,086.15	7%
Overhead costs	$ 1,686.41	6%
Mentoring visits by mentors (transport and meals)	$ 1,338.67	5%
Data review and prioritization sessions	$ 205.36	1%
**Grand Total**	**$ 27,955.74**	**100%**

According to the previously reported results from the evaluation of MESH-IMCI by Magge et al, the proportion of observed patients who were correctly diagnosed improved from 56% to 92%, and the proportion of observed patients who were correctly treated improved from 78% to 98% after 12 months of mentorship through MESH intervention [19]. The additional costs of MESH-IMCI for these improvements was US$1.06 per visit (Table 2).

**Table 2:** Incremental cost-effectiveness ratio for improved IMCI quality of care in a simulated cohort of 1000 patients.

	Baseline	Mentorship (12 months)	Difference
Median cost per visit	$0.18	$1.24	**$1.06**
Modeled N	1000	1000	
Total cost per visit for cohort	$176.63	$1,237.17	$1,060.54
*Classification*			
Correctly diagnosed (%)	56%	92%	
Modeled correctly diagnosed	560	920	360
C/E ratio (Difference in cost/difference in modeled correctly diagnosed)			**$2.95**
*Treatment*			
Correctly treated (%)	78%	98%	
Modeled correctly treated	780	980	200
C/E ratio (Difference in cost/difference in modeled correctly treated)			**$5.30**

We assumed no additional costs due to MESH-IMCI for improved care such as additional antibiotics or tests. Using our simulated population, we estimated the incremental cost per child correctly diagnosed was $2.95 and the incremental cost per child correctly treated was $5.30 (Table 2).

The actual improvements in quality of care did not depend on number of patient managed by the nurse per day. However, the incremental cost-effectiveness ratios were sensitive to changes in both volume of visits and baseline quality of care. As the number of children seen per IMCI-mentored nurses per day increased, the cost per child attributable to MESH-IMCI dropped. If the number of patients doubled, this cost dropped from $1.06 to $0.53, while if the number of patients was roughly increased from 26,360 to 79,080, this cost dropped to as low as $0.35. However, a lower number of children treated annually would increase the incremental cost added by the MESH intervention. For example, halving the actual number of children treated doubled the incremental cost from $1.06 to $2.12 (Table 3).

**Table 3:** Sensitivity analysis assuming lower or higher number of IMCI patients seen.

Scenario (volume)	Percentage higher and lower than actual number of patients	Number of children treated annually	Incremental cost added by MESH/ visit	Incremental C/E ratio per correct classification	ICER per correct treatment
Low (2)	-50%	13180	$2.12	5.89	10.61
Low (1)	-25%	19770	$1.41	3.93	7.07
Actual	0%	26360*	$1.06	2.95	5.30
High (1)	25%	32950	$0.85	2.36	4.24
High (2)	50%	39540	$0.71	1.96	3.54
High (3)	100%	52720	$0.53	1.47	2.65
High (4)	150%	65900	$0.42	1.18	2.12
High (5)	200%	79080	$0.35	0.98	1.77

*Actual total number of children treated according to IMCI protocols in the end-point year: HMIS 2012

Similarly, lower quality of care at baseline and greater improvement in diagnosis and treatment due to MESH-IMCI would decrease the incremental cost per child with correct diagnosis and treatment. For example, assuming a baseline level of 25% for correct diagnosis compared to 56% in our sample would result in a lower ICER ($1.58 v. $2.95), and assuming baseline level of 40% at baseline for correct treatment compared to a baseline level of 78% would result in a lowering of ICER as well from $5.30 to $1.83. However, a high quality at baseline and endpoint would result in higher incremental cost (Table 4).

**Table 4:** Sensitivity analysis assuming lower or higher baseline quality of care than the actual scenario.

Scenario (Improvement)	Correctly diagnosed at baseline	Correctly diagnosed at end-point	ICER correctly diagnosed	Correct treated at baseline	Correctly treated at end-point	ICER correctly treated
Low (4)	0.5%		**$1.22**	0.5%		**$1.14**
Low (2)	15%		**$1.38**	20%		**$1.36**
Low (2)	25%		**$1.58**	40%		**$1.83**
Low (1)	40%		**$2.04**	60%		**$2.79**
**Actual**	**56%**	**92%**	**$2.95**	**78%**	**98%**	**$5.30**
High (1)	60%		**$3.31**	80%		**$5.89**
High (2)	75%		**$6.24**	85%		**$8.16**
High (3)	85%		**$15.15**	90%		**$13.26**
High (4)	90%		**$53.03**	95%		**$35.35**

## Discussion

We found that the addition of a mentoring intervention following training in IMCI resulted in modest increases in the costs per child correctly diagnosed and child correctly treated compared to standard care. This study provides the results of the important implementation outcome of cost to policy makers and program implementers seeking to improve the quality of facility-based IMCI. Our sensitivity analyses suggest that these costs would be lower in settings with lower baseline quality of care and/or higher patient volume. In contrast, the incremental cost added by MESH would be higher if the quality of care is already high and/or if health facilities have a low patient volume. However, these are likely not common scenarios especially in settings where the utilization of health services are constantly high with major gaps in quality of care. In our previous study, improvement was seen in nurses who had already undergone formal IMCI training, suggesting that additional costs due to MESH would provide additional benefit to the initial investment made in standard IMCI trainings. These results can inform discussions regarding the resources required to support IMCI-trained health staff who serve as the leading providers to children under-five children in resource-limited settings.

Human resources accounted for the majority of program expenses. Supervisors able to provide on-site and ongoing mentorship in IMCI did not exist in the current MOH system, so the IMCI mentors represented a new cadre introduced into the district system. Given that nearly 80% of the costs of MESH-IMCI were attributable to human resources, integrating MESH into existing supervision structures would dramatically reduce program costs. In this case, if rather than introducing a new cadre of workers, existing clinical supervisors were trained in QI and clinical mentorship and MESH activities were integrated into their supervisory roles, mentors’ salary would not contribute to overall costs. However, it is critical to review the roles and responsibilities of the existing supervisors to understand the capacity and impact of the addition of this mentoring approach. The addition may require removal of tasks that do not necessarily fall under existing supportive supervision activities. For example, in some countries, supervisors are assigned to perform more data collection and verification/audit visits than supportive supervision during their visits. Task-shifting of these types of activities to other team members such as data-focused district team members would be one option. These decisions should depend on the local context, priorities and the existing resources. However if there is a significant gap in quality, adding additional staff to provide effective supportive supervision may need to become a prioritized investment from the national and sub-national level as a critical step to achieve quality universal health coverage [31]. Logistics and off-site allowances (food, airtime and transport fees) for mentors and program management team also were included in the human resources category of expenses reflecting the amount of time mentors spend on mentorship with HC nurses, but if the mentorship was combined with existing supervision visits, these costs could also be decreased.

In order to strengthen QI practices as part of MESH and provide data for MESH supervision, a strong M&E system was implemented and represented the second highest cost category for the program. Resources required for MESH M&E included data officer time for data entry from the paper observation forms, time for M&E managers to coordinate the flow of paper checklists from mentors to data officers, and analyst time to produce quarterly reports. Integrating electronic data capture by mentors would reduce need for data entry and analysis, representing another opportunity to reduce costs while accelerating data availability. Integrating MESH M&E into the existing MoH’s Health Management Information System would also further decrease costs.

The incremental cost per correct treatment was higher than the cost per correct classification. This higher cost was due in part to the high levels of quality of treatment prescription at baseline leaving relatively smaller room for improvement.

Although MESH intervention sites had a growing number of IMCI service utilization, our previous evaluation found that IMCI nurses were comfortable with case management after MESH [32] and the quality of care remained consistently high [19]. However, the assumption that the increased number reduces ICER should be interpreted with caution as dramatic increases in patient volume may compromise the quality of care and therefore affect the overall cost-effectiveness of the intervention. Program implementers should ensure that health facilities have an appropriate ratio of staff/patient prior to and during MESH implementation.

A number of IMCI evaluations assessed changes in infant illness, deaths and disability adjusted life years averted [33]. The diagnosis and treatment measures used in the primary MESH IMCI were derived from those used in other studies of IMCI implementation [34–36]. While a number of studies described components and cost-effectiveness of IMCI implementation [4,37–39],other studies have focused solely on the costs of preparing for and delivering IMCI, rather than the costs of improving and sustaining quality. Our study represents new information on the costs to improve the quality of IMCI over the course of one-year, estimating the costs per changes in quality associated with a post-IMCI training mentorship intervention. District estimates of the costs of IMCI training and care delivery in Tanzania were $2.30 per child [4], while HC estimates in Uganda were $0.98 per child [37]. Our findings suggest that the cost of improving quality of care through the MESH intervention is roughly double the per-patient facility costs of the initial implementation of IMCI. Another cost-effectiveness study done in inpatient settings in Kenya found that improving quality of care by one percentage point increase across the average of fourteen IMCI measures was $0.79 per patient [40]. Although this study design, population and contexts differ from our study, both highlight that improving the quality of care requires more resources than the costs attributable to training, care providers and supplies alone.

This study has a number of limitations. First, our estimates are for cost-effectiveness related to process improvements in quality of under-5 care. We do not report on the impact of improving quality of IMCI care on changes in childhood mortality and so we cannot explore the potential incremental cost per disability-adjusted life year averted. Our analysis relies on a twelve month pre-post evaluation of quality of IMCI care. MESH has been integrated into the district quality improvement approach, and so the support has been ongoing, but we do not have objective (non-mentor-reported) measurement if the benefits were sustained beyond the 12 months. If the frequency of mentoring can be reduced over time, this would result in lower incremental costs. However, further evaluation would be needed to determine the implication of the reduced dose and its impact on sustainability of IMCI quality of care.

Furthermore, we did not include potential broader benefits to the HC and district on systems-level improvements related to the QI work done by IMCI mentors working with nurses at HCs, which could lead to lower cost estimates. Finally, MESH was designed to improve the quality of care provided by IMCI trained nurses. As such, we used the disaggregated method from provider perspective and included only costs related to MESH intervention. We did not have the resources for additional data collection to capture benefits (immediate and delayed) from the patient/care giver’s perspective. However, future research interventions should estimate the cost-effectiveness of the IMCI training and MESH from a societal perspective.

While IMCI resource allocation is often limited to initial didactic trainings, our study finds that the additional cost of adding post-training mentorship to IMCI implementation strategies in Rwanda is modest. Integrating this program into existing MoH structures could result in lower costs to the health system to improve under-5 care and potentially lead to further reductions in under-5 mortality in rural Rwanda and similar settings. As countries join the global coalition to accelerate access to universal health coverage [41,42], low cost approaches such as clinical mentoring and systems focused improvement interventions will play an important role in insuring that access to health care is not just universal, but that it is of the quality needed to reach the health-related Sustainable Development Goals.
